# Histaminergic System and Vestibular Function in Normal and Pathological Conditions

**DOI:** 10.2174/1570159X22666240319123151

**Published:** 2024-03-19

**Authors:** Brahim Tighilet, Jessica Trico, Emna Marouane, Andreas Zwergal, Christian Chabbert

**Affiliations:** 1 Aix Marseille Université-CNRS, Laboratoire de Neurosciences Cognitives, LNC UMR 7291, Marseille, Groupe de Recherche Vertige (GDR#2074), France;; 2 Normandie Université, UNICAEN, INSERM, COMETE, CYCERON, CHU Caen, 14000, Caen, France;; 3 Department of Neurology, LMU University Hospital, Munich, Germany;; 4 German Center for Vertigo and Balance Disorders, LMU University Hospital, Munich, Germany

**Keywords:** Vestibular system, histaminergic system, vestibular disorders, neuroinflammation, histaminergic drugs, vertigo

## Abstract

Most neurotransmitter systems are represented in the central and peripheral vestibular system and are thereby involved both in normal vestibular signal processing and the pathophysiology of vestibular disorders. However, there is a special relationship between the vestibular system and the histaminergic system. The purpose of this review is to document how the histaminergic system interferes with normal and pathological vestibular function. In particular, we will discuss neurobiological mechanisms such as neuroinflammation that involve histamine to modulate and allow restoration of balance function in the situation of a vestibular insult. These adaptive mechanisms represent targets of histaminergic pharmacological compounds capable of restoring vestibular function in pathological situations. The clinical use of drugs targeting the histaminergic system in various vestibular disorders is critically discussed.

## 1 INTRODUCTION

### 1.1 Anatomical and Functional Organization of the Vestibular System

**Fig. (1):**
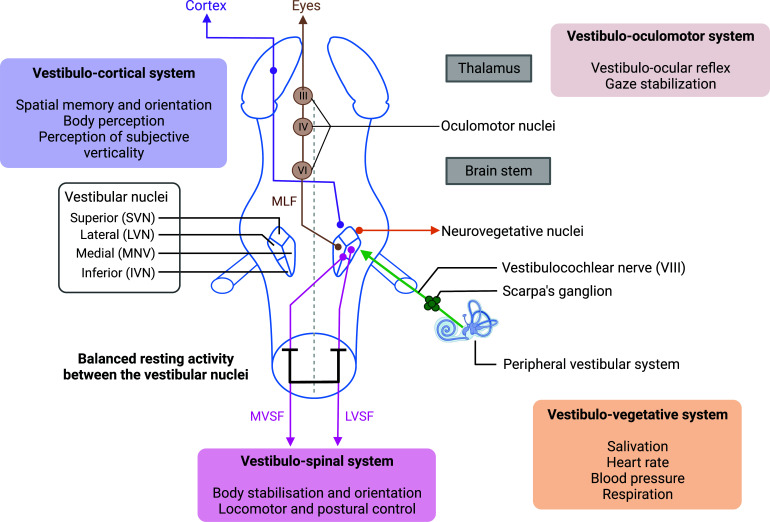
Anatomic and functional organization of the vestibular system. The vestibular nerve is in contact with the sensory hair cells located in the peripheral vestibular system. This nerve projects ipsilaterally to the four vestibular nuclei (VNs): superior, lateral, medial and inferior, located in the brainstem. From these nuclei, several output pathways exist. The vestibulo-oculomotor pathway originates from the lateral nuclei that project to the oculomotor nuclei *via* the medial longitudinal fasciculus (MLF), allowing the stabilization of gaze during head movements. The vestibulospinal pathway includes a lateral vestibulospinal fasciculus (LVSF) that connects the ipsilateral lateral VN to the spinal cord and a medial vestibulospinal fasciculus (MVSF) that connects the contralateral medial, inferior, and lateral VNs to the musculature of the neck and upper body axis. This organization establishes postural control and muscle tone. The vestibulo-vegetative pathway consists of the superior and medial VNs that activate the vagus nerve, responsible for vital functions. VNs are linked to several neurovegetative nuclei, such as the dorsal nucleus of the vagus nerve (DNV), solitary nucleus (NTS) and area postrema. Various vestibulo-cortical pathways originate from all VNs and project bilaterally to the cortex. The diversity of output from the VNs underlines the broad role of the vestibular system in posturo-locomotor, oculomotor and higher cognitive functions. The balanced resting activity between the bilateral VNs is crucial for these functions. Created with bioRender.

To maintain body's balance, the central nervous system uses visual, proprioceptive, tactile and above all, vestibular information. Vestibular inputs are detected by specific sensors located in the inner ear close to the cochlea. The vestibular sensors in each inner ear are organized in three cristae ampullaris and the two otolithic organs, the saccule and the utricle [1]. The crista ampullaris detects the accelerations resulting from angular movements, while the otolithic organs detect the accelerations resulting from linear movements of the head and gravitational accelerations. The sensory inputs generated by the vestibular sensors are conveyed through the vestibular nerve towards the first central nervous relay: the brainstem vestibular nuclei complex (VNC). This nuclear complex consists of four different vestibular nuclei (VN): the median vestibular nuclei (MVN), the inferior vestibular nuclei (IVN), the lateral vestibular nuclei (LVN) and the superior vestibular nuclei (SVN). The VNs are located in the dorsolateral part of the pontomedullary junction of the brainstem, under the floor of the fourth ventricle. The vestibular sensory information reaching the VN level is then integrated and converted into a specific motor message dedicated to the regulation and control of postural, locomotor balance and oculomotor function [2]. Hence this sensorimotor system designation is attributed exclusively to the vestibular system. The vestibulo-ocular reflex (VOR) intends essentially for stabilizing the gaze during head movements, and the vestibulo-spinal reflex (VSR) directs readjustments and stabilization of the head and body in static (standing) and dynamic (walking) conditions. These compensatory vestibular reflexes are exerted at the level of the extrinsic musculature of the eye through the vestibulo-ocular reflex, as well as at the level of the neck and trunk axial musculature, and limbs proximo-distal musculature through the vestibulo-spinal reflexes (Fig. **1**).

A particularity of this integrating nerve center is that the VNs receive a multitude of information from other sensory modalities such as vision, proprioception, touch and to a lesser degree hearing [3-7]. The vestibular system is also involved in perceptual and cognitive functions supported by cortical areas involved in the processing of peripheral vestibular inputs. Among these areas, well documented in the literature, the parieto-insular vestibular cortex is the most representative [8-11]. This multisensory area manages functions such as perception of verticality, representation of the body scheme and orientation of body in space [11-13]. The vestibular system also sends information to important nervous structures such as the hippocampus, the hypothalamus, the amygdala, the ventral tegmental area [14-19]. This is why VNs were recently referred as a “hub” linked to several networked nervous structures, receiving, organizing, and redirecting the sensory inputs to multiple functional targets [6, 20].

### 1.2 Unilateral Peripheral Vestibular Syndrome and its Compensation

**Fig. (2):**
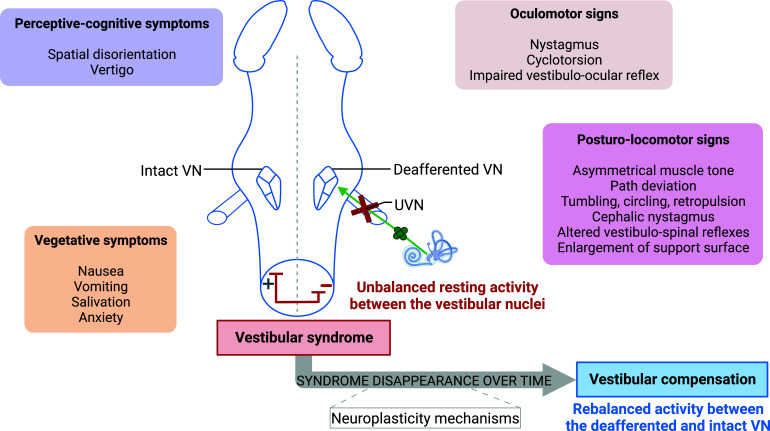
Unbalanced resting activity within the vestibular nuclei complex and vestibular syndrome expression after unilateral vestibular neurectomy. Electrophysiological imbalance after unilateral vestibular neurectomy (UVN) between the bilateral vestibular nuclei (VNs) is responsible for the acute vestibular syndrome. After UVN, the ipsilateral VNs are deafferented and show reduced excitability in contrast to the VNs contralateral to the lesion. This effect is explained by the absence of vestibular peripheral inputs from the lesion side. The syndrome generated by this unbalanced resting activity in the VNs is composed of oculomotor, posturo-locomotor, vegetative and perceptive-cognitive signs and symptoms. Over time, the syndrome disappears as neuroplasticity mechanisms result in a rebalanced activity between the bilateral VNs - a mechanism called vestibular compensation. Created with bioRender.

In human and most species, acute unilateral vestibular loss induces static and dynamic vestibular signs and symptoms consisting of vestibulo-ocular (nystagmus, cyclotorsion, altered vestibulo ocular reflex generating oscillopsia), posturo-locomotor (postural instability, falls, ipsilateral deviation from the walking path), vegetative (nausea, vomiting, salivation) and perceptive-cognitive (spatial disorientation, vertigo) deficits. These static and dynamic deficits compensate differently, either partially or completely and with different time course. This spontaneous post-lesional functional recovery is referred in the literature as “central vestibular compensation” (Fig. **2**) [21-23]. The static deficits (those present in absence of body movements) compensate for the most part. It is now well established that static deficits result from the imbalance of spontaneous resting activity between the ipsi- and contralesional VNs. Compensation approximately coincides with restoration of balanced electrical activity between VNCs. These events were confirmed electrophysiologically in many studies [24-26]. The dynamic deficits (those present during body movements) are not completely compensated and present for a longer time. Recovery of dynamic deficits seems not to depend on rebalanced activity in the VNs solely, but is attributed to multiple plasticity mechanisms occurring in various brain areas [21, 23, 27-35].

## 2 HISTAMINE AND VESTIBULAR FUNCTION

A large variety of neurotransmitters is present in the vestibular system. However, histamine occupies a peculiar position in vestibular physiology due to the expression of all histamine receptors (HR) in the vestibular sensory network. Histamine is also involved in regulating both the normal response to vestibular stimulation and the reactive processes that support vestibular compensation. Pharmacological modulation of histamine production and release has been proven to enhance functional recovery in vestibular animal models [36-38] and patients with vestibular disorders [39]. Thus, histamine receptors seem suitable targets for modulating vestibular sensory information.

### 2.1 Brain Source of Histamine

**Fig. (3):**
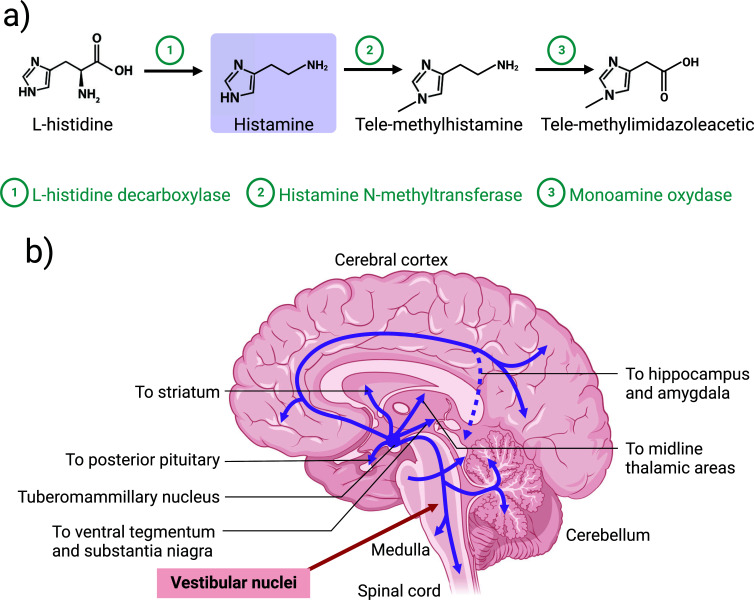
Synthesis, catabolism, and distribution of histamine in the adult mammalian brain. (**a**) Neuronal histamine is synthetized from the amino acid L-histidine by the L-histidine decarboxylase enzyme. Histamine is then degraded by two enzymes, histamine N-methyltransferase and monoamine oxidase, resulting in tele-methylimidazoleacetic. (**b**) Histaminergic neurons are restricted to the tuberomammillary nucleus of the posterior hypothalamus, from where they project widely into the brain, including in the vestibular nuclei complexes. Created with bioRender.

Histamine is synthesized from the amino acid L-histidine by the enzyme histidine decarboxylase and the main pathway of histamine degradation is based on the action of histamine N-methyltransferase followed by monoamine oxidase B [40] (Fig. **3a**). Brain sources of histamine include mast cells, neurons and microglia [40-42]. In the adult mammalian brain, the histaminergic neurons are exclusively located in the tuberomammillary nucleus of the posterior hypothalamus (TMN) from where they project their fibers and terminals to the whole brain including VNs (Fig. **3b**) [40, 43-46].

### 2.2 Brain Distribution of Histamine Receptors

Histaminergic receptors are widely found in the central nervous system.

Histamine type 1 receptor (H1R) exhibits a wide distribution in the central nervous system, particularly in brain areas involved in arousal, including the thalamus, cortex, cholinergic nuclei, locus coeruleus and raphe nuclei [40, 47, 48]. These findings have been ascertained through binding assays [49] and RT-PCR techniques [50] in rat models, autoradiography in ginea pigs [51] and *in situ* hybridation and receptor binding autoradiography [52], or PET-scan imaging [53] in human samples. Moreover, H1R has been located in the limbic system (*i.e*. several nuclei of the hypothalamus, medial amygdala and hippocampus) [40, 47] using binding assay techniques across diverse mammalian species [48, 54]. Receptor autoradiography techniques carried out on both human and non-human primate samples [55], alongside human PET-scan imaging [53], have consistently revealed the presence of H1R in the limbic system. Additionally, H1R is detectable in the cerebellum [53] and basal ganglia [55]. Nevertheless, it is worth knowing that interspecies variations exist [48, 54].

Given that both H1R and H2R are present in the same cerebral regions, an overlapping of their functions in behavior has been established [56]. H2R, akin to H1R, is localized in the hippocampus, amygdala, basal ganglia and cerebral cortex [40, 43]. These findings have been corroborated through several techniques, including *in situ* hybridization techniques in rat models [57], radioligand binding and *in situ* hybridization techniques in ginea pigs [58], as well as receptor binding autoradiography [55, 59] and *in situ* hybridization techniques [52] in non-human primates and humans samples. Finally, H2R is also localized in the cerebellum and hypothalamus [59].

H3R shares common localization with H1R and H2R. High-density expression of H3R is observed in the basal ganglia (*i.e*. striatum and substantia nigra), as well as the hippocampus [40, 47, 60]. These localizations have been ascertained through various techniques applied in rodent models, such as immunohistochemistry [61], receptor binding autoradiography [62] and *in situ* hybridization [63]. In human and non-human primate studies, H3R has been found in the basal ganglia using *in situ* hybridization and receptor binding techniques [64] while its presence in the hippocampus has been established using autoradiography [55]. Additionally, H3R has also been identified in the hypothalamus, thalamus and cortex [40, 52, 60, 61].

H4R has been localized in various regions of the rat central nervous system, including the cortex, cerebellum, brainstem, amygdala, thalamus and striatum [65]. Furthermore, H4R has been detected in human samples within the hippocampus, cortex, thalamus and amygdala [65, 66]. It is noteworthy that discrepancies in the distribution of H4R within the brain exist among different species [65] and the expression and function of H4R in the central nervous system remains controversial and requires further investigation [67].

### 2.3 Expression of Histaminergic Receptors in the Vestibular System

**Fig. (4):**
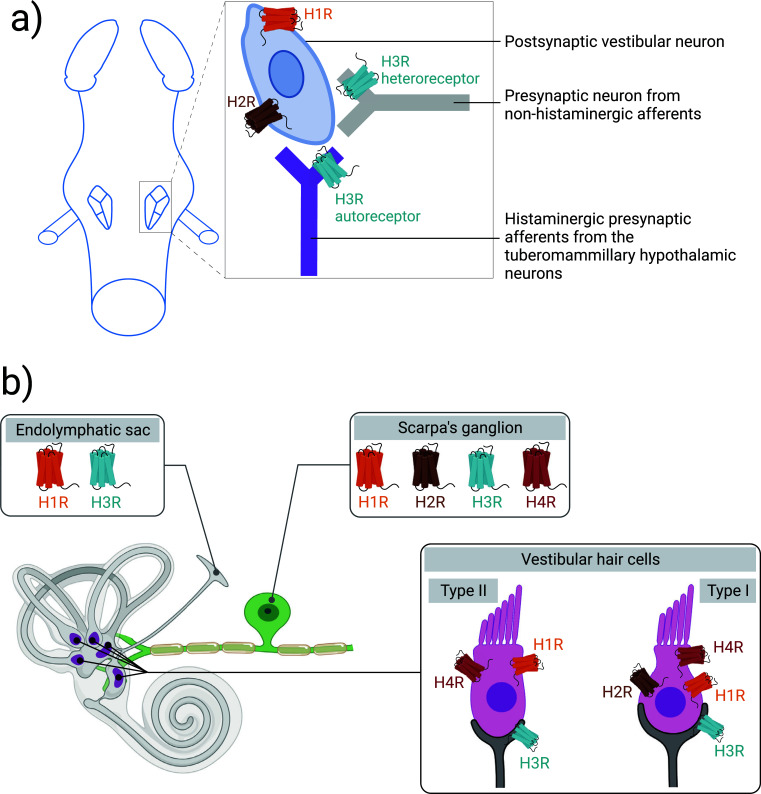
Localization of histaminergic receptors in the central (**a**) and peripheral (**b**) vestibular system. (**a**) The vestibular nuclei complexes (VNCs) contain three types of histaminergic receptors (HR). The secondary vestibular neurons express histaminergic type 1 (H1R) and histaminergic type 2 (H2R) receptor while the afferents from the tuberomammillary histaminergic neurons (in purple) express the histaminergic type 3 (H3R) autoreceptor. It should be noted that other afferents releasing various neurotransmitter in the VNCs can express a histaminergic type 3 (H3R) heteroreceptor. (**b**) The peripheral vestibular system contains all types of histaminergic receptors. Every type of HR is expressed in the Scarpa’s ganglion while only H1R and H3R are expressed in the endolymphatic sac. H1R, H3R and H4R are expressed in both type II and type I vestibular hair cells, while H2R is only found in type I vestibular hair cells. These schematic representations were created based on information found in [36, 58, 69] for the upper part and [71-73] for the lower part. Created with biorender.

The different types of histaminergic receptors are expressed at the levels of central and peripheral vestibular systems. At the central level (Fig. **4a**), the VNs contain three types of histamine receptors (H1R, H2R and H3R), as shown using ligand-binding [51, 58, 68, 69], *in situ* hybridization methods [58, 63, 70], or in immunohistochemistry [36]. H1R and H2R are located in a post-synaptic position in vestibular neurons, whereas H3R is located in a pre-synaptic position on histaminergic afferents from neurons of the posterior hypothalamus [62]. In this situation, H3R acts as an autoreceptor which regulates the synthesis and release of histamine. In addition, H3R can also be found on non-histaminergic afferents and is then considered as a heteroreceptor that modulates the regulation and synthesis of other neurotransmitters. Finally, as H4R is expressed on microglia, known to be present in VNs, the presence of H4R in VNs is highly probable (this aspect is developed in section 4). These aspects confer a neuromodulatory role to histamine in the brain.

In the peripheral labyrinth (Fig. **4b**), the four types of histamine receptors (H1R, H2R, H3R and H4R) have been located in both hair cells and vestibular primary neurons [71-74]. Using an RT-PCR approach, Takumida and colleagues recently revealed that the different types of histamine receptors are expressed in different proportions depending on the cell type considered. Vestibular hair cells are classified into two categories, distinguished by their morphology (bottle-shaped for type I vestibular hair cells and more cylindrical for type II vestibular hair cells), their ion channel equipment (g_K,L_ current is specific to type I vestibular hair cells) as well as their afferentation by vestibular nerve fibers (calyx endings are found exclusively on type I vestibular hair cells) [75]. According to Møller *et al*. combined microarrays and immunohistochemistry approaches showed expression of the H1R in the epithelial lining of the endolymphatic sac, while H3R are expressed exclusively in the subepithelial capillary network. H2R and H4R were not found in the endolymphatic sac [76].

### 2.4 Function of the Histamine Receptors in the Vestibular System

Despite ongoing research efforts, the physiological relevance of histamine receptors expression in the vestibular system remains unclear and requires further investigation.

At the peripheral level, it is known for almost fifty years that the antagonization of H1R (*i.e*., pyrilamine, diphenhydramine) significantly alters the vestibular primary neuron excitability in a dose dependent manner [77]. Similarly, H3R antagonists (*i.e*., thioperamide, clobenpropit and betahistine) decrease the electrical discharge of primary vestibular neurons [78] and betahistine could reduce resting activation rate of peripheral vestibular sensors [79].

At central level, *in vitro* extracellular and intracellular recordings in IVN, SVN, MVN and LVN neurons revealed histamine induced depolarization through postsynaptic H1R and H2R [80-86]. These findings attest that histamine has an excitatory action in the VNs.

Vestibular stimulations of rotatory [87], electric and caloric [88], or gravitational [89] nature commonly increase histamine release in the hypothalamus and brainstem. Local perfusion of the VN on one side with H2R antagonists or H3R agonists induces a stereotyped postural and oculomotor syndrome in the guinea pig that mimics that observed after labyrinthectomy [90].

Given the presence of histaminergic receptors throughout the peripheral and central vestibular system and their proven involvement in vestibular signal processing, there is a high likelihood that histamine also plays a significant role in vestibular compensation.

## 3 HISTAMINE AND VESTIBULAR COMPENSATION

**Fig. (5):**
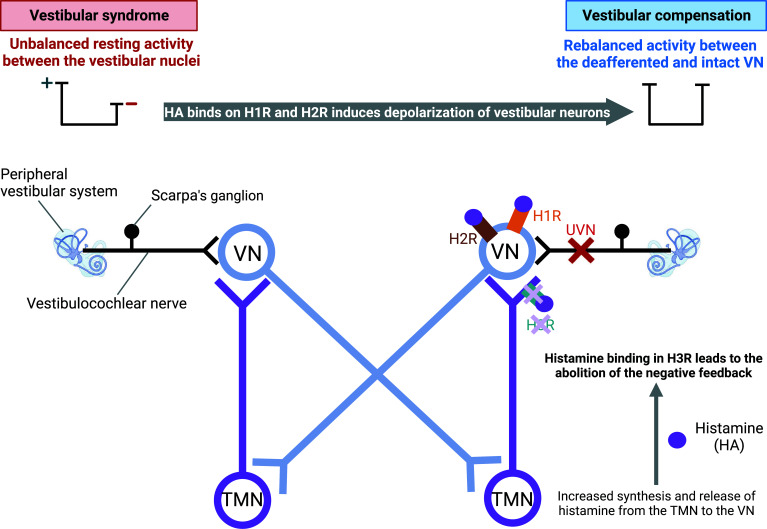
Involvement of histamine in vestibular compensation after unilateral vestibular neurectomy. The unilateral vestibular neurectomy (UVN) leads to an electrophysiological imbalance between the vestibular nuclei, which is conveyed to the posterior hypothalamus through direct vestibulo-hypothalamic loops. In consequence, the synthesis and release of histamine (HA) from the tuberomammillary nucleus (TMN) to the deafferented vestibular nuclei (VN) increases. In VN, HA will bind to the three types of histamine receptors. By the histamine H3 receptor (H3R), more HA will be synthesized and released because of the abolition of the negative feedback. By the histamine H1 (H1R) and H2 (H2R) receptors, HA will restore the electrophysiological imbalance underlying the vestibular compensation. Created with bioRender.

A direct link between vestibular compensation and increased histamine turnover has been established in the central nervous system of adult cats [37, 38, 69, 91, 92]. Unilateral section of the vestibular nerve induces a spontaneous electrical activity imbalance between the bilateral vestibular VNs with hyperactivity on the intact side and hypoactivity on the deafferented side. This electrophysiological imbalance induces an activation of histaminergic tuberomammillary neurons of the posterior hypothalamus *via* vestibulo-hypothalamic loops [93] which results both in a local increase of the histamine synthesis and its release in the VNs (Fig. **5**). These mechanisms are prevented upon bilateral vestibular neurectomy [69]. This confirms that the electrophysiological asymmetry between the intact and deafferented VN is the cause of the histaminergic system activation. The elevated histamine release may participate in rebalancing the spontaneous activity between the bilateral VNs, a key parameter of the vestibular compensation, by its depolarizing effect on H1R and H2R (Fig. **5**). Furthermore, altered histamine levels after a vestibular injury may have effects on various vestibular plasticity mechanisms, such as neuroinflammation.

## 4 EFFECT OF HISTAMINE ON NEUROINFLAMMATION IN VESTIBULAR DISORDERS

Inflammation is a multi-faced process which involves complex cellular and molecular mechanisms triggered by stress, injury, or infection, with the ultimate goal of returning to physiological homeostasis. In the central nervous system, inflammation is known as neuroinflammation and is characterized by the involvement of two key players: microglial cells and astrocytes, which, under physiological conditions, contribute to the central nervous system homeostasis.

### 4.1 Neuroinflammation in Acute Peripheral Vestibulopathy

Acute unilateral vestibulopathy (AUVP; vestibular neuritis), characterized by vertigo/dizziness, spontaneous nystagmus, postural imbalance and vegetative symptoms, has been associated with the presence of local and systemic inflammation in affected patients [94, 95]. Although its etiology is still debated (viral, vascular, inflammatory), inflammation of the vestibular afferents (labyrinth/vestibular nerve) is one of the proposed causes [96] and has led to the administration of corticosteroids as a treatment principle [97]. While data from clinical trials suggest some effect of early administration of corticosteroids on the recovery of peripheral vestibular function on the longer term [98], its benefit for vestibular compensation is questioned by recent meta-analyses [97, 99-101]. Accordingly, there is consensus that further drug research is needed to better control the acute symptoms of patients with AUVP.

### 4.2 Neuroinflammation in Animal Models of Acute Peripheral Vestibulopathy

Some animal models of vestibular loss generate an inflammatory response in the central nervous structures which process vestibular information. Postganglionic damage to vestibular afferents by unilateral vestibular neurectomy (UVN) results in the recruitment of astrocytes [102-105] and microglia [104, 105] in the ipsilesional VNs. Given its ability to consistently reproduce the typical behavioral phenotype of AUVP, the UVN model is particularly relevant to study the role of central inflammation in vestibular pathophysiology and to investigate how pharmacological modulation of inflammation can affect the expression of this pathology. Other models of vestibulopathies are known to generate central vestibular inflammation, such as the surgical labyrinthine destruction model [103, 106-108] or chemical model using arsanilate [109]. Transtympanic injection of arsanilate irreversibly desensitizes the vestibular sensors of the inner ear [110]. This chemical vestibular lesion model induces the expression of two key inflammatory factors, the tumor necrosis factor-alpha (TNF-α) and the nuclear factor-kappa B (NF-kB) in the deafferented VNs [109]. Furthermore, this model elicits both a peripheral (vestibular nerve) and central (VNC) inflammatory response [111]. Thus, all these models are appropriate to study the impact of neuroinflammatory processes in vestibular core hubs. The interest in understanding the role of inflammatory processes correlated with vestibular pathologies goes well beyond the vestibular models mentioned above since pro-inflammatory signatures have also been recently reported in Menière's disease and vestibular migraine [112].

### 4.3 The Dual Role of Histamine in Neuroinflammation

Astrocytes and microglial cells are crucial components of the glial inflammatory response in the central nervous system [113]. These cells express histamine receptors [114, 115] and microglial cells produce histamine [41]. The idea that histamine would play a role in inflammation has thus encouraged much research. Xu and al. demonstrated that histamine inhibits the production of the pro-inflammatory cytokine TNF-α and interleukin 1 beta (IL-1β) in a concentration-dependent manner in astrocytes. In addition, a knockout mouse model of the histamine-synthesizing enzyme, histidine decarboxylase, led to a decrease in microglial arborization [116]. Thus, there is a strong link between histamine and inflammation, but this link is complex. Multiple studies have demonstrated the dual role of histamine in modulating inflammation depending on the microenvironment [117, 118].

*In vitro* and *in vivo* histaminergic stimulation of H1R and H4R results in the activation of microglia, which then secretes proinflammatory factors such as TNF-α, interleukin 6 (IL-6), IL-1β, prostaglandin E2 and reactive oxygen species (ROS) [114, 119-122]. Furthermore, activation of H1R also elicits an increase in phagocytic activity [120]. The proinflammatory response elicited by histamine administration can result in neuronal damage and even neuronal degeneration, both *in vitro* and *in vivo* [120, 123].

**Fig. (6):**
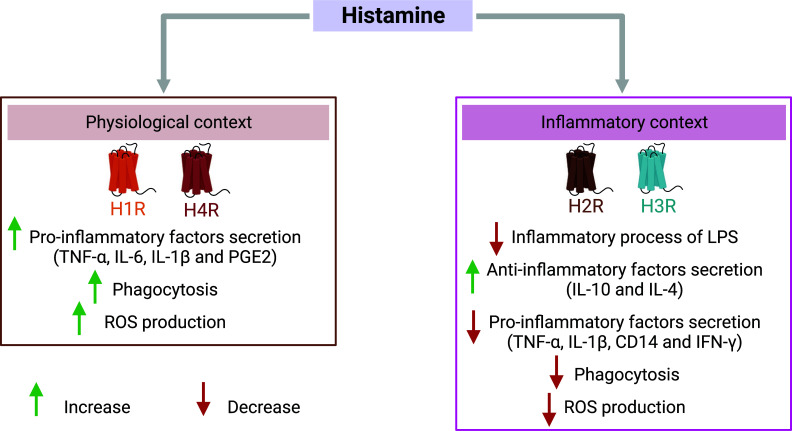
Dual role of histamine depending on the context of the cellular environment. Histamine has a rather pro-inflammatory or anti-inflammatory role *via* the four histamine receptors expressed by microglia, depending on the micro-environment. Histamine has a pro-inflammatory role *via* the histamine H1 (H1R) and H4 (H4R) in a physiological context. Their activation leads to increased secretion of pro-inflammatory factors such as tumor necrosis factor-alpha (TNF-α), interleukin 6 (IL-6), interleukin 1 beta (IL-1β) and prostaglandin E2 (PGE2), but also to increased phagocytosis and reactive oxygen species (ROS) production. Conversely, histamine has a rather anti-inflammatory role in an inflammatory context *via* the histamine H2 (H2R) and H3 (H3R) receptors. Their activation leads to the inhibition of the inflammatory process of lipopolysaccharide (LPS), to the increase of secretion of anti-inflammatory factors such as interleukin 10 (IL-10) and interleukin 4 (IL-4), to the inhibition of pro-inflammatory factors (TNF-α and IL-1β), cluster of differentiation 14 (CD14) and interferon-gamma (IFN-γ). Finally, activation of these receptors also decreases phagocytosis and ROS production. Created with bioRender.

Contrary to its proinflammatory effect in a physiological context, histamine has been demonstrated to possess anti-inflammatory properties in an inflammatory micro-environment (Fig. **6**). The anti-inflammatory action of histamine is primarily mediated by H2R and H3R, which trigger the release of anti-inflammatory cytokines such as interleukin 10 (IL-10) [121, 124, 125]. *In vitro* studies have shown that histaminergic stimulation of H2R suppresses inflammation induced by lipopolysaccharide (LPS) injection in human monocytes by decreasing TNF-α secretion [126] and cluster of differentiation 14 (CD14) expression [127]. Similar effects have been observed in human monocyte-derived dendritic cells and immune cells, where histamine increased the production of anti-inflammatory cytokines (IL-4), reduced the production of pro-inflammatory factors (TNF-α, d’IFN-γ) and led to cytoskeleton rearrangements [128]. Additionally, after LPS injection, histamine modulated microglial cells *in vitro* by reducing migration and IL-1β release [118] and dose-dependently inhibited phagocytosis and cytokine production (TNF-α) through H3R activation [129]. The anti-inflammatory effects of histamine have also been established *in vivo*, where it has been showed to inhibit phagocytic activity and ROS production, contributing to a neuroprotective effect [117]. The relationship between the histaminergic system and neuroinflammation is thus robust and underscores the neuroprotective nature of histamine in an inflammatory context.

### 4.4 Neuroinflammation and Vestibular Compensation

**Fig. (7):**
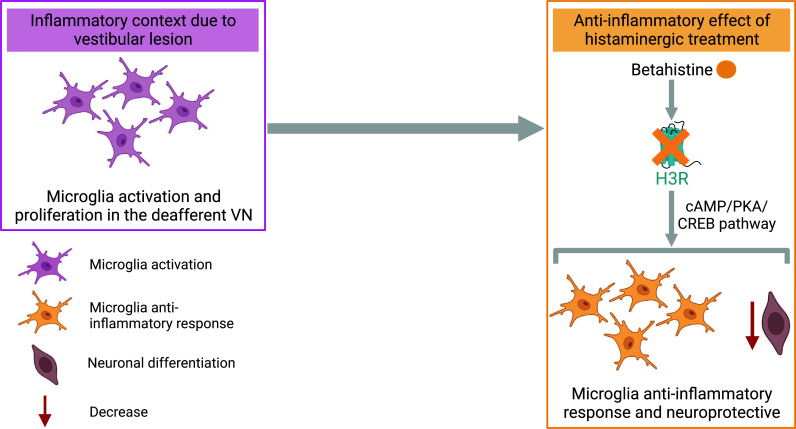
Anti-inflammatory and neuroprotective effect of betahistine after vestibular lesion. The vestibular lesion induces an inflammatory context in the deafferented vestibular nuclei (VN). Betahistine inhibits the histamine H3 receptor (H3R), which leads to the activation of a cAMP/PKA/CREB signaling pathway that induces glial-mediated inhibition of inflammation. These elements lead to a neuroprotective environment and would explain the decrease of neuronal differentiation in deafferented VN under this treatment. Created with bioRender.

Microglial and astrocytic responses are expressed at their peak during the acute phase of the vestibular syndrome and then gradually decrease over time, but persist during the chronic phase [103, 105, 106, 108, 130]. A recent longitudinal study utilizing advanced imaging and tracing techniques has revealed that a vestibular lesion activates microglia in the vestibular nerve and brainstem nuclei [111]. Thus, a vestibular lesion induces an inflammatory context in the VNs that plays a crucial role in vestibular compensation and functional recovery. There is a delicate balance between inflammation and vestibular compensation, since an anti-inflammatory treatment during the acute phase of the vestibular syndrome delays both vestibular compensation and adaptive plasticity [131]. However, the relationship between inflammation and vestibular compensation is complex. Indeed, sensorimotor rehabilitation [132] and pharmacological treatments such as L-thyroxine [133] or betahistine (unpublished data) have been shown to increase the differentiation and survival of microglial cells at the expense of neurons, and reduce significantly posturo-locomotor deficits in an animal model of vestibulopathy. This surprising result, in view of the demonstration of functional reactive neurogenesis after vestibular injury [102, 105], could be explained by a microglial anti-inflammatory neuroprotective action, conferred by the effective rehabilitation or pharmacological treatment used. Moreover, a preliminary study has shown differential expression dynamics of microglial phenotypes during vestibular compensation [134]. Further studies on the amount of pro- or anti-inflammatory cytokines in these contexts are thus interesting. In betahistine-treated UVN animals, this anti-inflammatory effect may be mediated by H3R blockade, leading to activation of the cAMP/PKA/CREB pathway and the inhibition of glial-mediated inflammation [135]. This hypothesis is supported by the observation that histamine prevents the reduction of CREB protein levels only in an inflammatory context [136]. Thus, elevated histamine levels may promote differentiation towards a microglial phenotype with a neuroprotective role in a neuroinflammatory context triggered by vestibular injury. This neuroprotective phenotype could help to reduce the number of dying neurons, thus avoiding the cost of neuronal differentiation to the organism (Fig. **7**). Further studies on the rate of apoptosis and cytokine levels in VN as a function of rehabilitation or pharmacological therapy would be of great interest. The central role of inflammation in vestibular disorders is further supported by a recent study in Menière’s disease patients, where elevated levels of IL-1β and TNF-α were observed [137]. All these data highlight a modulation of vestibular functions by central histaminergic system and suggest potential targets for clinical treatment of vestibular disorders.

## 5 HISTAMINE RECEPTORS MODULATORS AND PHARMACOTHERAPY OF VESTIBULAR DISORDERS

### 5.1 Differential Applications of Histaminergic Drugs in Various Vestibular Disorders

The pharmacotherapy of the vestibular system is relatively well documented [72, 138, 139], but an effective treatment for the diverse symptoms of vestibular syndromes remains to be found. Histamine receptor modulation holds a central place in drug research on vestibular disorders, given the abundant expression of HRs across the peripheral and central vestibular networks. Modulators of the H1R and H3R are among the drugs most commonly used in the treatment of vestibular disorders [72, 140-142], while H4R modulators are under development in this field [71, 143]. From a conceptual perspective, HR modulators could be helpful in the following clinical scenarios, each of which require a slightly different mode of action based on the underlying pathophysiological mechanisms: 1) application as an antivertigo drug in episodes of vestibular imbalance by suppression of peripheral or central vestibular tone asymmetry, 2) reduction of attack frequency and duration in hydroptic ear disease (including Menière’s disease) by action on fluid homeostasis in the inner ear, 3) use for augmentation of central vestibular compensation following AUVP by modulation of adaptive neuroplasticity mechanism.

### 5.2 Treatments Based on Histamine Receptor Type 1 Antagonization (Antivertigo Drugs)

Besides betahistine, there are a few drugs in clinical use for symptom control in vestibular disorders, which convey at least some of their therapeutic effects by antagonism to histamine receptors:

*Dimenhydrinate* predominently acts *via* antagonism to the H1R and muscarinergic acetylcholine receptor (mAChR): [144, 145]. Functionally, dimenhydrinate suppresses spontaneous as well as stimulation-induced firing of neurons in the vestibular nuclei [146]. It is considered as a vestibuar suppressant and frequently used for the symptomatic treatment of nausea and vomiting in acute episodes of vertigo or dizziness or the prevention of motion sickness.

*Meclizine* and *cyclizine* are antagonists at H1R, but in addition have anticholinergic effects. They are in use in the United States for the treatment of nausea, vomiting in vestibular disorders and motion sickness. Their antiemetic and antivertigo effects are not fully understood, but its central antihistaminergic and anticholinergic properties are likely involved. The drugs depress labyrinth excitability and vestibular stimulation and may affect the medullary chemoreceptor trigger zone.

*Cinnarizine* has multiple actions, including blockage of L-/T-type voltage-gated calcium channels and antagonism to the H1R [147]. It is also used for symptomatic treatment of vertigo, nausea, vomiting and motion sickness in clinical practice. Cinnarizine acts as a vestibular suppressant mostly on peripheral vestibular structures by inhibition of Ca^2+^ ion translocation across cell membranes of the vestibular sensory cells in the ampullae [148], by modulation of transmitter release in vestibular hair cells [149], and potentially also by anti-vasoconstrictive action in the stria vascularis [150]. Some binding of cinnarizine to voltage-gated calcium channels in the cerebral cortex has been reported [151]. Convincing experimental data for a direct receptor-mediated central action at the level of the vestibular nuclei is however missing [152].

Cinnarizine is also available in fixed combination with dimenhydrinate in various countries for the indications vertigo, nausea and vomiting in acute and episodic vestibular disorders [153-155]. In a prospective study, *cinnarizine/dimenhydrinate* (20 mg/40 mg) showed a non-inferiority to betahistine (16 mg) for symptomatic treatment of vertigo in patients with peripheral vestibular disorders [156, 157]. Observational studies also suggest an effect of either cinnarizine or cinnarizine/dimenhydrinate for attack reduction in vestibular migraine [158, 159].

*Flunarizine* is a first-generation antihistaminergic drug, which acts as an antagonist to the H1R. In addition, it is a selective calcium entry blocker with calmodulin binding properties. Thus, it has pharmacological similarities to cinnarizine. Main indications in vestibular medicine are the symptomatic treatment of peripheral and central vertigo [160] as well as vestibular migraine [161, 162].

In summary, vestibular suppressants such as the first-generation anihistaminergic durgs dimenhydrinate, meclizine, cyclizine, cinnarizine or flunarizine have a reverse binding pattern to H1R compared to betahistine. In consequence, the major difference of their action may be that the H1R antagonists suppress symptoms, while having the risk to delay mechanisms of central vestibular plasticity after unilateral peripheral vestibular loss. In contrast, betahistine does have less acute symptomatic effects, but a positive action for adaptive vestibular neuroplasticity (developped in section 5.5). Further, H1R antagonists but not the H1R agonist betahistine tend to have sedative side effects such as drowsiness.

### 5.3 Treatments Based on Histamine Receptor in the Inner Ear (Hydroptic Ear Disease)

In terms of the pharmacological treatment of Menière disease, betahistine has remained the most widely used drug in Europe for several decades [72, 140, 163, 164]. Experimental data from animal models give a strong rationale for a positive action of betahistine in the inner ear. At the peripheral vascular system level, betahistine has been shown to increase cochlear and vestibular blood flows, improving the inner ear microcirculation [165-168]. This effect is believed to be mediated by histamine H3 heteroreceptors and H1 receptors [169-172] which could alleviate the microcirculation impairments and decrease the endolymphatic hydrops accompanying Menière’s disease [165, 170].

Nevertheless, the clinical literature differs on the betahistine efficacy [173]. No effect of betahistine treatment has been found at low or high dose in Menière’s disease patients in a double blind, randomized and placebo controlled trial [174], while others studies found positive effect of betahistine treatment in vestibular disorders [39, 175-179]. There is a need for more thorough research on the effectiveness of betahistine in patients suffering from Menière’s disease [180]. Future studies should use the consented diagnostic criteria, select drug formulations, which take the specific metabolism of betahistine into account, always include a control group, and apply meaningful patient-oriented outcome measures.

### 5.4 Treatments Based on Augmentation of Vestibular Compensation by Histamine Receptor Modulation (AUVP)

Preclinical research on the use of betahistine in animal models of acute unilateral vestibular deafferentation is limited. The few reported works show a beneficial action of an oral treatment with betahistine on the acute vestibular syndrome in a cat [37, 38, 181] and rodent UVN model (unpublished data). Another study highlights that the faster recovery in posturo-locomotor symptoms in cats following UVN occurs in conjunction with elevated plasma concentrations of betahistine and an upregulation of histidine decarboxylase in the hypothalamus [38]. In the rodent model of surgical labyrinthectomy, either microinjection of betahistine in MVNs, intragastric infusion of betahistine or continuous administration through an osmotic minipump had beneficial effects on the restauration of vestibular functions [36, 182, 183]. In a chemical labyrinthectomy rat model, betahistine had dose-dependent beneficial effects on postural imbalance and mobility (Antons *et al*., 2023). Surgical vestibulopathy models, such as UVN and labyrinthectomy, as well as the unilateral intratympanic gentamycin application model [184] are comparable as they all lead to similar vestibular symptoms [185]. These lesions induce a vestibular syndrome similar to AUVP encountered in the clinic, characterized by sudden onset of vertigo or dizziness, accompanied by nausea and vomiting, gait instability, head motion intolerance and nystagmus, which persist over at least a day. Besides the sporadic cases of AUVP (*i.e*., vestibular neuritis), surgical or chemical damage to the peripheral vestibular system is also used sporadically to treat vestibular disorders in humans (*e.g*., in vestibular schwannoma, intractable Menière’s disease) [186-188]. However, a recently published prospective controlled trial comparing the intranasal application of betahistine against placebo in patients undergoing vestibular neurectomy failed to show significant group effects [189].

Given these considerations, it appears that there are still some problems with translating positive preclinical experimental data into the clinical setting, which may be explained by the special betahistine mode of action and metabolism. The mechanisms are outlined in the following chapters.

### 5.5 Betahistine Mechanism of Action

As evoked above, betahistine has the potential to modulate peripheral and central vestibular networks and thus improve pathophysiology of various vestibular disorders based on its specific mode of action.

#### 5.5.1 Antagonistic Action on Histamine H3 Autoreceptors and Heteroreceptors

Betahistine is a partial H1R agonist and H3R antagonist. Different mechanisms account for the effect of betahistine on the restoration of vestibular function. It is now well established that histamine H3 autoreceptor mediates autoinhibition of brain histamine release and autoregulation of histamine synthesis [43, 190-193]. The histamine H3 heteroreceptor mediates autoinhibition of neurotransmitters such as glutamate, GABA, acetylcholine, norepinephrine, dopamine, serotonin, and peptides [40, 194, 195]. These neurotransmitters are all located in the VNs and play a role in regulating vestibular functions and the compensation process [72].

Through blocking the histamine H3 autoreceptors, betahistine increases the synthesis and release of histamine in the TMN and the VN, as shown by immunohistochemistry, *in situ* hybridization and binding to H3R methods and improves vestibular compensation in the UVN cat model [37, 38, 68, 91, 92, 196]. Thus, betahistine may increase histamine release, which may bind to H1R and H2R in the VN, leading to depolarization which may participate in restoring balanced activity between the deafferented and intact VN.

#### 5.5.2 Agonistic Action on Histamine H1 Receptors

**Fig. (8):**
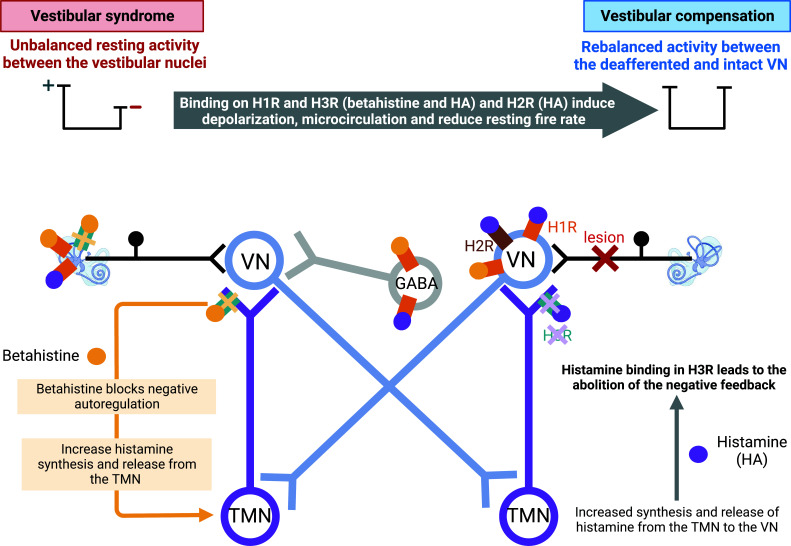
Pharmacological mechanisms of betahistine underlying the rebalanced activity between the deafferented and intact vestibular nuclei after a vestibular lesion. Betahistine increases histamine (HA) synthesis and secretion by blocking histamine H3 (H3R) auto-receptors. Several events participate in the restoration of vestibular functions. Histaminergic activation of histamine H1 receptor (H1R) by betahistine and histamine and of histamine H2 receptor (H2R) by histamine in excitatory neurons of the deafferented vestibular nuclei (VN) induces depolarization. Betahistine and histamine bind to H1R on ipsilesional commissural GABAergic neurons, inhibiting the VN contralateral to the lesion. Peripherally, betahistine could increase the cochlear and vestibular blood flow, improving the microcirculation of the inner ear *via* histamine H3 heteroreceptors, while activation of H1R by betahistine and histamine induces a reduction in the activity of intact vestibular inputs. Created with bioRender.

Betahistine can restore electrophysiological balance between the VNCs either by histamine depolarization action on central vestibular neurons expressing H1R or H2R or by agonistic action on H1R located on vestibular excitatory neurons on the deafferented side. This electrophysiological balance is necessary for the vestibular compensation (Fig. **8**).

Betahistine has also been demonstrated to restore electrophysiological balance between homologous VN and promotes a faster vestibular compensation by reducing activity in the contralateral VN through its agonistic effect on H1R located on inhibitory GABAergic vestibular commissural neurons in the deafferented MVN [36]. This H1 receptor-mediated asymmetric activation of the vestibular commissural inhibitory system participate in rebalancing the two medial vestibular nuclei and contributes to recovery of both static and dynamic symptoms in locomotion and motor coordination.

In addition to this central mechanism of action, betahistine could reduce both vestibular receptors and afferent vestibular neurons resting firing rate leading to a decrease in the sensory input from the vestibular endorgans [72, 79, 197, 198]. This effect of betahistine on the intact side may restore an electrophysiological homeostasis between the two peripheral vestibular systems. Betahistine could also restore the homeostasis of endolymphatic fluid by acting on H3R and H1R [76, 170]. This could be a way to reduce the increased pressure observed in the endolymphatic hydrops, which is considered as the pathophysiological correlate of Menière's disease [170].

#### 5.5.3 Action of Betahistine on Histaminergic Glial Receptors Leading to Restauration of Excitability

Betahistine could enhance excitability in the deafferented vestibular environment by an action on histamine receptors carried by glial cells and thus restore an electrophysiological balance between the two VNCs. It is well known that astrocytes and microglial cells play a crucial role in regulating brain circuit activity through dynamic interactions with neurons. Astrocytes can modulate neuronal network excitability thanks to different processes, such as K+ clearance [199] or intracellular Ca^2+^ elevations regulating release of gliotransmitters and neuronal excitability [200]. The expression of H1R, H2R, and H3R has been identified on astrocytes [201]. Histamine interacts with the H1R expressed by astrocytes and leads to the release of glutamate in a concentration-dependent manner in cell cultures [202]. This mechanism increasing glutamate concentration might occur in the deafferented VNs, leading to a higher excitability thus enhancing the restoration of activity between the two VNCs and vestibular compensation. Moreover, H1R regulates the glutamate clearance to avoid the excitotoxic effect [203]. Microglia also controls neuronal network excitability *via* BDNF signaling [204]. Furthermore, microglia is involved in the oligodendrocyte progenitor cell homeostasis and the myelin components [205], which are capital for the excitability. Since microglial cells express all H1R, H2R, H3R and H4R [114], the increase in histamine promoted by betahistine could intervene on these key mechanisms in order to restore an electrophysiological balance and thus improve vestibular compensation.

#### 5.5.4 Clinical use of Betahistine

Previous clinical studies on the use of betahistine in various vestibular disorders often neglected its specific pharmacokinetic properties when applied *via* an oral route. Betahistine undergoes extensive first-pass metabolism and therefore has short biological half-life (3-4 hours). The lack of efficacy of betahistine in vestibular disorders could be due to its fast metabolism, given that 95% of the drug is rapidly metabolized by the monoamine oxidase a/b (MAO). Furthermore, genetic polymorphism may cause different rates of metabolism across patients. To increase efficacy in patients with high metabolism, the daily dose may need to be higher and/or the treatment duration longer. Combination of betahistine with a MAO inhibitor (such as selegiline or rasagiline) could also improve its effectiveness in patients with vestibular disorders, as the plasma level of betahistine may rise up by factor 100 (unpublished data). In fact, a low betahistine dose (0.2 mg/kg) co-administrated with selegiline (1 mg/kg) accelerated balance recovery in UVN animals similarly to a single-drug treatment with a 10-times higher betahistine dose (2 mg/kg) [38]. This data strongly supports that the betahistine effect is dependent on its plasma bioavailability. Taken together, co-administration of betahistine with selegiline could be a promising pharmacological therapy both for patients with Menière's disease and AUVP. Changing the route of administration could also be an interesting track given the presence of high concentration of MAO in the digestive tract. One possibility would be to administer betahistine through the nasal route, which would bypass the digestive system and reduce its metabolism. This new administration route has been recently tested in a double-blind clinical study [189].

Finally, pharmacokinetic studies have shown that betahistine is transformed into aminoethylpyridine and hydroxyethylpyridine at the hepatic level and is then excreted as pyridylacetic acid in urine. It was shown that aminoethylpyridine was able to reduce the resting discharge of ampullar receptors like betahistine [206]. In addition, aminoethylpyridine and hydroxyethylpyridine, were found to induce similar effects on the micro-circulation than betahistine in the guinea pig model [165]. This might be of some clinical interest. Based on these data, the anti-vertigo action of betahistine may initially be achieved by the drug itself and then sustained by its metabolites. A therapeutic perspective would be to use betahistine as well as its metabolites.

## 6 FUTURE DEVELOPMENT OF HISTAMINE-RECEPTOR MODULATORS

### 6.1 Histamine H4 Receptor Modulators

Following the demonstration of a preferred expression of H4R in rodent vestibular primary neurons [71], selective H4R antagonists have been found to have a strong inhibitory effect on the excitability of both isolated mammalian vestibular primary neurons [71] and rodent vestibule explants [74]. Since H3R antagonist used in pharmacology, such has betahistine, share this peripheral effect [78], these results suggested a potential application of H4R antagonists in vestibular disorders.

Recently, a selective inhibitor of the H4R, SENS-111, was given orally to healthy volunteers who underwent caloric tests. This administration significantly improved the latency of vertigo appearance, disappearance and duration, as well as the European Evaluation of Vertigo questionnaire parameters compared to baseline [143]. Although the drug had no significant effect on nystagmus, this study suggests that SENS-111 may be a promising drug for managing dizziness associated with vestibular disorder. Another recent investigation in Menière’s disease patients reported that a particular H4R gene variant, rs77485247 polymorphism, may be linked to an increased risk of Menière’s disease [207]. H4R are highly expressed on peripheral blood mononuclear cells [208], which are critical components of the immune system. There is growing evidence of an autoimmune background for Menière’s disease in a subset of patients [209] and that proinflammatory cytokines contribute to pathogenesis of Menière’s disease [137]. These findings suggest a connection between inflammation, histamine receptors, and vestibular disorders.

### 6.2 Histamine, Neuroinflammation and H4 Receptor

As mentioned above, microglial cells, which are the innate immune cells in the central nervous system and primarily express H4R [67], can be activated by histamine, resulting in the production of pro-inflammatory cytokines [114]. This observation is supported by data showing that intracerebroventricular infusion of H4R agonists increased the total microglia cell number in a dose-dependent manner [116]. In a rat model of Parkinson’s pathology, Zhou and al, showed that H4R antagonist inhibits pro-inflammatory microglia response and prevents the progression of Parkinson-like pathology and behavior [210]. Given the presence of H4R in microglial cells and the high level of expression of these cells in the VN following vestibular loss, it can be postulated that histamine may play a role in the inflammatory response observed in this model of vestibulopathy. Therefore, the use of H4R antagonist pharmacological compounds may be appropriate to inhibit the inflammation associated with certain vestibular pathologies, such as AUVP.

## CONCLUSION

As evidenced by the wide expression of its various specific receptors throughout the vestibular sensory network, histamine appears to be a major modulator of vestibular signal processing. However, its precise actions on the detection, transmission and integration of vestibular sensory information in normal physiological conditions, which could globally favor the activation of the vestibule in case of need, still remain to be specified. The fine interactions between the histaminergic system and the inflammation modulating cells reveal a neuroprotective action of histamine, which could be valuable in an inflammatory context associated with peripheral vestibular damage. In addition, the extensive histamine receptor expression across the peripheral and central vestibular network offers a large opportunity for pharmacological modulation of the central compensation process with the hope of optimizing functional restoration of peripheral vestibular loss. Current efforts to improve the delivery of pharmacological compounds that have already proven their efficacy on the symptoms of vestibular disorders should soon lead to more targeted and effective drugs for vestibular pathologies.

## LIST OF ABBREVIATIONS

H1R: Histamine Type 1 Receptor
HR: Histamine Receptors
IVN: Inferior Vestibular Nuclei
LVN: Lateral Vestibular Nuclei
MVN: Median Vestibular Nuclei
NF-kB: Nuclear Factor-kappa B
SVN: Superior Vestibular Nuclei
TNF-α: Tumor Necrosis Factor-alpha
UVN: Unilateral Vestibular Neurectomy
VN: Vestibular Nuclei
VNC: Vestibular Nuclei Complex
VOR: Vestibulo-ocular Reflex
VSR: Vestibulo-spinal Reflex

## References

[1] Kingma H., van de Berg R. (2016). Anatomy, physiology, and physics of the peripheral vestibular system.. Handb. Clin. Neurol..

[2] Angelaki D.E., Cullen K.E. (2008). Vestibular system: The many facets of a multimodal sense.. Annu. Rev. Neurosci..

[3] Azzena G.B., Mameli O., Tolu E. (1980). Distribution of visual input to the vestibular nuclei.. Arch. Ital. Biol..

[4] Cullen K.E. (2012). The vestibular system: Multimodal integration and encoding of self-motion for motor control.. Trends Neurosci..

[5] Gdowski G.T., McCrea R.A. (2000). Neck proprioceptive inputs to primate vestibular nucleus neurons.. Exp. Brain Res..

[6] MacKinnon C.D. (2018). Sensorimotor anatomy of gait, balance, and falls.. Handb. Clin. Neurol..

[7] McCall A.A., Miller D.M., DeMayo W.M., Bourdages G.H., Yates B.J. (2016). Vestibular nucleus neurons respond to hindlimb movement in the conscious cat.. J. Neurophysiol..

[8] Brandt T., Dieterich M. (2019). Thalamocortical network: A core structure for integrative multimodal vestibular functions.. Curr. Opin. Neurol..

[9] Waele C., Baudonniere P.M., Lepecq J.C., Huy P.T.B., Vodal P.P. (2001). Vestibular projections in the human cortex.. Exp. Brain Res..

[10] Dieterich M., Brandt T. (2018). The parietal lobe and the vestibular system.. Handb. Clin. Neurol..

[11] Lopez C., Blanke O. (2011). The thalamocortical vestibular system in animals and humans.. Brain Res. Brain Res. Rev..

[12] Lopez C. (2015). Making sense of the body: The role of vestibular signals.. Multisens. Res..

[13] Lopez C. (2016). The vestibular system.. Curr. Opin. Neurol..

[14] Brandt T., Schautzer F., Hamilton D.A., Brüning R., Markowitsch H.J., Kalla R., Darlington C., Smith P., Strupp M. (2005). Vestibular loss causes hippocampal atrophy and impaired spatial memory in humans.. Brain.

[15] Brandt T., Dieterich M. (2020). ‘Excess anxiety’ and ‘less anxiety’: Both depend on vestibular function.. Curr. Opin. Neurol..

[16] Cullen K.E. (2019). Vestibular processing during natural self-motion: implications for perception and action.. Nat. Rev. Neurosci..

[17] Jacob P.Y., Poucet B., Liberge M., Save E., Sargolini F. (2014). Vestibular control of entorhinal cortex activity in spatial navigation.. Front. Integr. Nuerosci..

[18] McKeown J., McGeoch P.D., Grieve D.J. (2020). The influence of vestibular stimulation on metabolism and body composition.. Diabet. Med..

[19] Smith P.F. (2022). Recent developments in the understanding of the interactions between the vestibular system, memory, the hippocampus, and the striatum.. Front. Neurol..

[20] Tighilet B., Chabbert C. (2019). Adult neurogenesis promotes balance recovery after vestibular loss.. Prog. Neurobiol..

[21] Curthoys I.S. (2000). Vestibular compensation and substitution.. Curr. Opin. Neurol..

[22] Curthoys I.S., Halmagyi G.M. (1995). Vestibular compensation: A review of the oculomotor, neural, and clinical consequences of unilateral vestibular loss.. J. Vestib. Res..

[23] Lacour M., Helmchen C., Vidal P.P. (2016). Vestibular compensation: The neuro-otologist’s best friend.. J. Neurol..

[24] Smith P.F., Curthoys I.S. (1988). Neuronal activity in the ipsilateral medial vestibular nucleus of the guinea pig following unilateral labyrinthectomy.. Brain Res..

[25] Smith P.F., Curthoys I.S. (1988). Neuronal activity in the contralateral medial vestibular nucleus of the guinea pig following unilateral labyrinthectomy.. Brain Res..

[26] Zennou-Azogui Y., Borel L., Lacour M., Ez-Zaher L., Ouaknine M. (1993). Recovery of head postural control following unilateral vestibular neurectomy in the cat. Neck muscle activity and neuronal correlates in Deiters’ nuclei.. Acta Otolaryngol..

[27] Antons M., Lindner M., Eilles E., Günther L., Delker A., Branner C., Krämer A., Beck R., Oos R., Wuehr M., Ziegler S., Strupp M., Zwergal A. (2023). Dose- and application route-dependent effects of betahistine on behavioral recovery and neuroplasticity after acute unilateral labyrinthectomy in rats.. Front. Neurol..

[28] Darlington C.L., Smith P.F. (2000). Molecular mechanisms of recovery from vestibular damage in mammals: Recent advances.. Prog. Neurobiol..

[29] Dieringer N. (1995). ‘Vestibular compensation’: neural plasticity and its relations to functional recovery after labyrinthine lesions in frogs and other vertebrates.. Prog. Neurobiol..

[30] Dutia M.B. (2010). Mechanisms of vestibular compensation: Recent advances.. Curr. Opin. Otolaryngol. Head Neck Surg..

[31] Grosch M., Lindner M., Bartenstein P., Brandt T., Dieterich M., Ziegler S., Zwergal A. (2021). Dynamic whole-brain metabolic connectivity during vestibular compensation in the rat.. Neuroimage.

[32] Lacour M. (2006). Restoration of vestibular function: Basic aspects and practical advances for rehabilitation.. Curr. Med. Res. Opin..

[33] Lacour M., Tighilet B. (2010). Plastic events in the vestibular nuclei during vestibular compensation: The brain orchestration of a “deafferentation” code.. Restor. Neurol. Neurosci..

[34] Paterson J.M., Short D., Flatman P.W., Seckl J.R., Aitken A., Dutia M.B. (2006). Changes in protein expression in the rat medial vestibular nuclei during vestibular compensation.. J. Physiol..

[35] Smith P.F., Curthoys I.S. (1989). Mechanisms of recovery following unilateral labyrinthectomy: A review.. Brain Res. Brain Res. Rev..

[36] Chen Z.P., Zhang X.Y., Peng S.Y., Yang Z.Q., Wang Y.B., Zhang Y.X., Chen X., Wang J.J., Zhu J.N. (2019). Histamine H1 receptor contributes to vestibular compensation.. J. Neurosci..

[37] Tighilet B., Mourre C., Trottier S., Lacour M. (2007). Histaminergic ligands improve vestibular compensation in the cat: Behavioural, neurochemical and molecular evidence.. Eur. J. Pharmacol..

[38] Tighilet B., Léonard J., Watabe I., Bernard-Demanze L., Lacour M. (2018). Betahistine treatment in a cat model of vestibular pathology: Pharmacokinetic and pharmacodynamic approaches.. Front. Neurol..

[39] Redon C., Lopez C., Bernard-Demanze L., Dumitrescu M., Magnan J., Lacour M., Borel L. (2011). Betahistine treatment improves the recovery of static symptoms in patients with unilateral vestibular loss.. J. Clin. Pharmacol..

[40] Brown R.E., Stevens D.R., Haas H.L. (2001). The physiology of brain histamine.. Prog. Neurobiol..

[41] Katoh Y., Niimi M., Yamamoto Y., Kawamura T., Morimoto-Ishizuka T., Sawada M., Takemori H., Yamatodani A. (2001). Histamine production by cultured microglial cells of the mouse.. Neurosci. Lett..

[42] Schwartz J.C., Arrang J.M., Garbarg M., Pollard H., Ruat M. (1991). Histaminergic transmission in the mammalian brain.. Physiol. Rev..

[43] Haas H., Panula P. (2003). The role of histamine and the tuberomamillary nucleus in the nervous system.. Nat. Rev. Neurosci..

[44] Panula P., Yang H.Y., Costa E. (1984). Histamine-containing neurons in the rat hypothalamus.. Proc. Natl. Acad. Sci. USA.

[45] Panula P., Pirvola U., Auvinen S., Airaksinen M.S. (1989). Histamine-immunoreactive nerve fibers in the rat brain.. Neuroscience.

[46] Tighilet B., Lacour M. (1996). Distribution of histaminergic axonal fibres in the vestibular nuclei of the cat.. Neuroreport.

[47] Hu W., Chen Z. (2017). The roles of histamine and its receptor ligands in central nervous system disorders: An update.. Pharmacol. Ther..

[48] Chang R.S.L., Tran V.T., Snyder S.H. (1979). Heterogeneity of histamine H1-receptors: species variations in [3H]mepyramine binding of brain membranes.. J. Neurochem..

[49] Bárbara A., Aceves J., Arias-Montaño J.A. (2002). Histamine H1 receptors in rat dorsal raphe nucleus: Pharmacological characterisation and linking to increased neuronal activity.. Brain Res..

[50] Korotkova T.M., Sergeeva O.A., Ponomarenko A.A., Haas H.L. (2005). Histamine excites noradrenergic neurons in locus coeruleus in rats.. Neuropharmacology.

[51] Bouthenet M.L., Ruat M., Sales N., Garbarg M., Schwartz J.C. (1988). A detailed mapping of hist amine H1-receptors in guinea-pig central nervous system established by autoradiography with [125I]iodobolpyramine.. Neuroscience.

[52] Jin C.Y., Panula P. (2005). The laminar histamine receptor system in human prefrontal cortex suggests multiple levels of histaminergic regulation.. Neuroscience.

[53] Yanai K., Tashiro M. (2007). The physiological and pathophysiological roles of neuronal histamine: An insight from human positron emission tomography studies.. Pharmacol. Ther..

[54] Kanba S., Richelson E. (1984). Histamine H1 receptors in human brain labelled with [3H]Doxepin.. Brain Res..

[55] Martinez-Mir M.I., Pollard H., Moreau J., Arrang J.M., Ruat M., Traiffort E., Schwartz J.C., Palacios J.M. (1990). Three histamine receptors (H1, H2 and H3) visualized in the brain of human and non-human primates.. Brain Res..

[56] Schneider E.H., Neumann D., Seifert R. (2014). Modulation of behavior by the histaminergic system: Lessons from H1R-and H2R-deficient mice.. Neurosci. Biobehav. Rev..

[57] Karlstedt K., Senkas A., Åhman M., Panula P. (2001). Regional expression of the histamine H2 receptor in adult and developing rat brain.. Neuroscience.

[58] Vizuete M.L., Traiffort E., Bouthenet M.L., Ruat M., Souil E., Tardivel-Lacombe J., Schwartz J.C. (1997). Detailed mapping of the histamine H2 receptor and its gene transcripts in guinea-pig brain.. Neuroscience.

[59] Traiffort E., Pollard H., Moreau J., Ruat M., Schwartz J.C., Martinez-Mir M.I., Palacios J.M. (1992). Pharmacological characterization and autoradiographic localization of histamine H2 receptors in human brain identified with [125I]iodoaminopotentidine.. J. Neurochem..

[60] Yoshikawa T., Nakamura T., Yanai K. (2021). Histaminergic neurons in the tuberomammillary nucleus as a control center for wakefulness.. Br. J. Pharmacol..

[61] Chazot P.L., Hann V., Wilson C., Lees G., Thompson C.L. (2001). Immunological identification of the mammalian H3 histamine receptor in the mouse brain.. Neuroreport.

[62] Pollard H., Moreau J., Arrang J.M., Schwartz J.C. (1993). A detailed autoradiographic mapping of histamine H3 receptors in rat brain areas.. Neuroscience.

[63] Pillot C., Heron A., Cochois V., Tardivel-Lacombe J., Ligneau X., Schwartz J.C., Arrang J.M. (2002). A detailed mapping of the histamine H3 receptor and its gene transcripts in rat brain.. Neuroscience.

[64] Anichtchik O.V., Peitsaro N., Rinne J.O., Kalimo H., Panula P. (2001). Distribution and modulation of histamine H(3) receptors in basal ganglia and frontal cortex of healthy controls and patients with Parkinson’s disease.. Neurobiol. Dis..

[65] Strakhova M.I., Nikkel A.L., Manelli A.M., Hsieh G.C., Esbenshade T.A., Brioni J.D., Bitner R.S. (2009). Localization of histamine H4 receptors in the central nervous system of human and rat.. Brain Res..

[66] Shan L., Bossers K., Luchetti S., Balesar R., Lethbridge N., Chazot P.L., Bao A.M., Swaab D.F. (2012). Alterations in the histaminergic system in the substantia nigra and striatum of Parkinson’s patients: a postmortem study.. Neurobiol. Aging.

[67] Schneider E.H., Seifert R. (2016). The histamine H4-receptor and the central and peripheral nervous system: A critical analysis of the literature.. Neuropharmacology.

[68] Tighilet B., Trottier S., Mourre C., Chotard C., Lacour M. (2002). Betahistine dihydrochloride interaction with the histaminergic system in the cat: Neurochemical and molecular mechanisms.. Eur. J. Pharmacol..

[69] Tighilet B., Trottier S., Mourre C., Lacour M. (2006). Changes in the histaminergic system during vestibular compensation in the cat.. J. Physiol..

[70] Ruat M., Traiffort E., Arrang J.M., Leurs R., Schwartz J.C. (1991). Cloning and tissue expression of a rat histamine H2-receptor gene.. Biochem. Biophys. Res. Commun..

[71] Desmadryl G., Gaboyard-Niay S., Brugeaud A., Travo C., Broussy A., Saleur A., Dyhrfjeld-Johnsen J., Wersinger E., Chabbert C. (2012). Histamine H 4 receptor antagonists as potent modulators of mammalian vestibular primary neuron excitability.. Br. J. Pharmacol..

[72] Soto E., Vega R. (2010). Neuropharmacology of vestibular system disorders.. Curr. Neuropharmacol..

[73] Takumida M., Takumida H., Anniko M. (2016). Localization of histamine (H1, H2, H3 and H4) receptors in mouse inner ear.. Acta Otolaryngol..

[74] Wersinger E., Gaboyard-Niay S., Travo C., Soto E., Baez A., Vega R., Brugeaud A., Chabbert C. (2013). Symptomatic treatment of vestibular deficits: Therapeutic potential of histamine H4 receptors.. J. Vestib. Res..

[75] Eatock R.A., Songer J.E. (2011). Vestibular hair cells and afferents: Two channels for head motion signals.. Annu. Rev. Neurosci..

[76] Møller M.N., Kirkeby S., Vikeså J., Nielsen F.C., Caye-Thomasen P. (2016). Expression of histamine receptors in the human endolymphatic sac: The molecular rationale for betahistine use in Menieres disease.. Eur. Arch. Otorhinolaryngol..

[77] Housley G.D., Norris C.H., Guth P.S. (1988). Histamine and related substances influence neurotransmission in the semicircular canal.. Hear. Res..

[78] Chávez H., Vega R., Soto E. (2005). Histamine (H3) receptors modulate the excitatory amino acid receptor response of the vestibular afferents.. Brain Res..

[79] Botta L., Mira E., Valli S., Perin P., Zucca G., Valli P. (1998). Effects of betahistine on vestibular receptors of the frog.. Acta Otolaryngol..

[80] Li B., Zhang X-Y., Yang A-H., Peng X.C., Chen Z.P., Zhou J.Y., Chan Y.S., Wang J.J., Zhu J.N. (2017). Histamine increases neuronal excitability and sensitivity of the lateral vestibular nucleus and promotes motor behaviors *via* hcn channel coupled to H2 receptor.. Front. Cell. Neurosci..

[81] Peng S.Y., Zhuang Q.X., He Y.C., Zhu J.N., Wang J.J. (2013). Histamine excites neurons of the inferior vestibular nucleus in rats by activation of H1 and H2 receptors.. Neurosci. Lett..

[82] Phelan K.D., Nakamura J., Gallagher J.P. (1990). Histamine depolarizes rat medial vestibular nucleus neurons recorded intracellularly *in vitro*.. Neurosci. Lett..

[83] Serafin M., Khateb A., Vibert N., Vidal P.P., Mühlethaler M. (1993). Medial vestibular nucleus in the guinea-pig: Histaminergic receptors. I. An *in vitro* study.. Exp. Brain Res..

[84] Wang J.J., Dutia M.B. (1995). Effects of histamine and betahistine on rat medial vestibular nucleus neurones: Possible mechanism of action of anti-histaminergic drugs in vertigo and motion sickness.. Exp. Brain Res..

[85] Zhang J., Han X.H., Li H.Z., Zhu J.N., Wang J.J. (2008). Histamine excites rat lateral vestibular nuclear neurons through activation of post-synaptic H2 receptors.. Neurosci. Lett..

[86] Zhuang Q.X., Wu Y.H., Wu G.Y., Zhu J.N., Wang J.J. (2013). Histamine excites rat superior vestibular nuclear neurons *via* postsynaptic H1 and H2 receptors *in vitro*.. Neurosignals.

[87] Takeda N., Morita M., Kubo T., Yamatodani A., Watanabe T., Wada H., Matsunaga T. (1986). Histaminergic mechanism of motion sickness. Neurochemical and neuropharmacological studies in rats.. Acta Otolaryngol..

[88] Horii A., Takeda N., Matsunaga T., Yamatodani A., Mochizuki T., Okakura-Mochizuki K., Wada H. (1993). Effect of unilateral vestibular stimulation on histamine release from the hypothalamus of rats *in vivo*.. J. Neurophysiol..

[89] Uno A., Takeda N., Horii A., Morita M., Yamamoto Y., Yamatodani A., Kubo T. (1997). Histamine release from the hypothalamus induced by gravity change in rats and space motion sickness.. Physiol. Behav..

[90] Yabe T., de Waele C., Serafin M., Vibert N., Arrang J.M., Mühlethaler M., Vidal P.P. (1993). Medial vestibular nucleus in the guinea-pig: Histaminergic receptors.. Exp. Brain Res..

[91] Tighilet B., Mourre C., Lacour M. (2014). Plasticity of the histamine H3 receptors after acute vestibular lesion in the adult cat.. Front. Integr. Nuerosci..

[92] Tighilet B., Lacour M. (1997). Histamine immunoreactivity changes in vestibular-lesioned and histaminergic-treated cats.. Eur. J. Pharmacol..

[93] Matsuyama T., Kayahara T., Nomura J., Nakano K. (1996). Direct projections from the medial vestibular nucleus to the posterior hypothalamic area in the monkey (Macaca fuscata).. Neurosci. Lett..

[94] Kassner S.S., Schöttler S., Bonaterra G.A., Stern-Straeter J., Hormann K., Kinscherf R., Gössler U.R. (2011). Proinflammatory activation of peripheral blood mononuclear cells in patients with vestibular neuritis.. Audiol. Neurotol..

[95] Strupp M., Bisdorff A., Furman J., Hornibrook J., Jahn K., Maire R., Newman-Toker D., Magnusson M. (2022). Acute unilateral vestibulopathy/vestibular neuritis: Diagnostic criteria.. J. Vestib. Res..

[96] Le T.N., Westerberg B.D., Lea J. (2019). Vestibular neuritis: Recent advances in etiology, diagnostic evaluation, and treatment.. Adv. Otorhinolaryngol..

[97] Goudakos J.K., Markou K.D., Franco-Vidal V., Vital V., Tsaligopoulos M., Darrouzet V. (2010). Corticosteroids in the treatment of vestibular neuritis: A systematic review and meta-analysis.. Otol. Neurotol..

[98] Strupp M., Zingler V.C., Arbusow V., Niklas D., Maag K.P., Dieterich M., Bense S., Theil D., Jahn K., Brandt T. (2004). Methylprednisolone, valacyclovir, or the combination for vestibular neuritis.. N. Engl. J. Med..

[99] Fishman J.M., Burgess C., Waddell A. (2011). Corticosteroids for the treatment of idiopathic acute vestibular dysfunction (vestibular neuritis).. Cochrane Libr..

[100] Kim G., Seo J.H., Lee S.J., Lee D.H. (2022). Therapeutic effect of steroids on vestibular neuritis: Systematic review and meta‐analysis.. Clin. Otolaryngol..

[101] Leong K.J., Lau T., Stewart V., Canetti E.F.D. (2021). Systematic review and meta‐analysis: Effectiveness of corticosteroids in treating adults with acute vestibular neuritis.. Otolaryngol. Head Neck Surg..

[102] Dutheil S., Brezun J.M., Leonard J., Lacour M., Tighilet B. (2009). Neurogenesis and astrogenesis contribution to recovery of vestibular functions in the adult cat following unilateral vestibular neurectomy: cellular and behavioral evidence.. Neuroscience.

[103] Dutheil S., Lacour M., Tighilet B. (2011). Neurogenic potential of the vestibular nuclei and behavioural recovery time course in the adult cat are governed by the nature of the vestibular damage.. PLoS One.

[104] Dutheil S., Watabe I., Sadlaoud K., Tonetto A., Tighilet B. (2016). BDNF signaling promotes vestibular compensation by increasing neurogenesis and remodeling the expression of potassium-chloride cotransporter KCC2 and GABAA receptor in the vestibular Nuclei.. J. Neurosci..

[105] Rastoldo G., El Mahmoudi N., Marouane E., Pericat D., Watabe I., Toneto A., López-Juárez A., Chabbert C., Tighilet B. (2021). Adult and endemic neurogenesis in the vestibular nuclei after unilateral vestibular neurectomy.. Prog. Neurobiol..

[106] Campos T.A., Vidal P.P., de Waele C. (1999). Evidence for a microglial reaction within the vestibular and cochlear nuclei following inner ear lesion in the rat.. Neuroscience.

[107] Campos-Torres A., Touret M., Vidal P.P., Barnum S., de Waele C. (2005). The differential response of astrocytes within the vestibular and cochlear nuclei following unilateral labyrinthectomy or vestibular afferent activity blockade by transtympanic tetrodotoxin injection in the rat.. Neuroscience.

[108] Waele C., Torres A.C., Josset P., Vidal P.P. (1996). Evidence for reactive astrocytes in rat vestibular and cochlear nuclei following unilateral inner ear lesion.. Eur. J. Neurosci..

[109] Liberge M., Manrique C., Bernard-Demanze L., Lacour M. (2010). Changes in TNFα, NFκB and MnSOD protein in the vestibular nuclei after unilateral vestibular deafferentation.. J. Neuroinflammation.

[110] Vignaux G., Chabbert C., Gaboyard-Niay S., Travo C., Machado M.L., Denise P., Comoz F., Hitier M., Landemore G., Philoxène B., Besnard S. (2012). Evaluation of the chemical model of vestibular lesions induced by arsanilate in rats.. Toxicol. Appl. Pharmacol..

[111] Zwergal A., Günther L., Brendel M. (2017). https://www.frontiersin.org/article/10.3389/fneur.2017.00665.

[112] Flook M., Frejo L., Gallego-Martinez A., Martin-Sanz E., Rossi-Izquierdo M., Amor-Dorado J.C., Soto-Varela A., Santos-Perez S., Batuecas-Caletrio A., Espinosa-Sanchez J.M., Pérez-Carpena P., Martinez-Martinez M., Aran I., Lopez-Escamez J.A. (2019). Differential proinflammatory signature in vestibular migraine and meniere disease.. Front. Immunol..

[113] Karve I.P., Taylor J.M., Crack P.J. (2016). The contribution of astrocytes and microglia to traumatic brain injury.. Br. J. Pharmacol..

[114] Dong H., Zhang W., Zeng X., Hu G., Zhang H., He S., Zhang S. (2014). Histamine induces upregulated expression of histamine receptors and increases release of inflammatory mediators from microglia.. Mol. Neurobiol..

[115] Xu J., Zhang X., Qian Q., Wang Y., Dong H., Li N., Qian Y., Jin W. (2018). Histamine upregulates the expression of histamine receptors and increases the neuroprotective effect of astrocytes.. J. Neuroinflammation.

[116] Frick L., Rapanelli M., Abbasi E., Ohtsu H., Pittenger C. (2016). Histamine regulation of microglia: Gene-environment interaction in the regulation of central nervous system inflammation.. Brain Behav. Immun..

[117] Barata-Antunes S., Cristóvão A.C., Pires J., Rocha S.M., Bernardino L. (2017). Dual role of histamine on microglia-induced neurodegeneration.. Biochim. Biophys. Acta Mol. Basis Dis..

[118] Ferreira R., Santos T., Gonçalves J., Baltazar G., Ferreira L., Agasse F., Bernardino L. (2012). Histamine modulates microglia function.. J. Neuroinflammation.

[119] Lenz K.M., Pickett L.A., Wright C.L., Davis K.T., Joshi A., McCarthy M.M. (2018). Mast cells in the developing brain determine adult sexual behavior.. J. Neurosci..

[120] Rocha S.M., Saraiva T., Cristóvão A.C., Ferreira R., Santos T., Esteves M., Saraiva C., Je G., Cortes L., Valero J., Alves G., Klibanov A., Kim Y.S., Bernardino L. (2016). Histamine induces microglia activation and dopaminergic neuronal toxicity *via* H1 receptor activation.. J. Neuroinflammation.

[121] Zhang W., Zhang X., Zhang Y., Qu C., Zhou X., Zhang S. (2020). Histamine induces microglia activation and the release of proinflammatory mediators in rat brain *via* H1R or H4R.. J. Neuroimmune Pharmacol..

[122] Zhu J., Qu C., Lu X., Zhang S. (2014). Activation of microglia by histamine and substance P.. Cell. Physiol. Biochem..

[123] Rocha S.M., Pires J., Esteves M. (2014). Histamine: A new immunomodulatory player in the neuron-glia crosstalk.. Front Cell Neurosci..

[124] Elenkov I.J., Webster E., Papanicolaou D.A., Fleisher T.A., Chrousos G.P., Wilder R.L. (1998). Histamine potently suppresses human IL-12 and stimulates IL-10 production *via* H2 receptors.. J. Immunol..

[125] Mazzoni A., Young H.A., Spitzer J.H., Visintin A., Segal D.M. (2001). Histamine regulates cytokine production in maturing dendritic cells, resulting in altered T cell polarization.. J. Clin. Invest..

[126] Morichika T., Takahashi H.K., Iwagaki H., Yoshino T., Tamura R., Yokoyama M., Mori S., Akagi T., Nishibori M., Tanaka N. (2003). Histamine inhibits lipopolysaccharide-induced tumor necrosis factor-alpha production in an intercellular adhesion molecule-1- and B7.1-dependent manner.. J. Pharmacol. Exp. Ther..

[127] Takahashi H.K., Morichika T., Iwagaki H., Tamura R., Kubo S., Yoshino T., Mori S., Akagi T., Tanaka N., Nishibori M. (2003). Histamine downregulates CD14 expression *via* H2 receptorson human monocytes.. Clin. Immunol..

[128] Aldinucci A., Bonechi E., Manuelli C., Nosi D., Masini E., Passani M.B., Ballerini C. (2016). Histamine regulates actin cytoskeleton in human toll-like receptor 4-activated monocyte-derived dendritic cells tuning CD4+ T lymphocyte response.. J. Biol. Chem..

[129] Iida T., Yoshikawa T., Matsuzawa T., Naganuma F., Nakamura T., Miura Y., Mohsen A.S., Harada R., Iwata R., Yanai K. (2015). Histamine H 3 receptor in primary mouse microglia inhibits chemotaxis, phagocytosis, and cytokine secretion.. Glia.

[130] Li H., Godfrey D.A., Rubin A.M. (1999). Astrocyte reaction in the rat vestibular nuclei after unilateral removal of Scarpa’s ganglion.. Ann. Otol. Rhinol. Laryngol..

[131] El Mahmoudi N., Rastoldo G., Marouane E., Péricat D., Watabe I., Tonetto A., Hautefort C., Chabbert C., Sargolini F., Tighilet B. (2021). Breaking a dogma: acute anti-inflammatory treatment alters both post-lesional functional recovery and endogenous adaptive plasticity mechanisms in a rodent model of acute peripheral vestibulopathy.. J. Neuroinflammation.

[132] Marouane E., El Mahmoudi N., Rastoldo G., Péricat D., Watabe I., Lapôtre A., Tonetto A., Xavier F., Dumas O., Chabbert C., Artzner V., Tighilet B. (2021). Sensorimotor rehabilitation promotes vestibular compensation in a rodent model of acute peripheral vestibulopathy by promoting microgliogenesis in the deafferented vestibular nuclei.. Cells.

[133] Rastoldo G., Marouane E., El-Mahmoudi N., Péricat D., Watabe I., Lapotre A., Tonetto A., López-Juárez A., El-Ahmadi A., Caron P., Fraysse M.J.E., Chabbert C., Zwergal A., Tighilet B. (2022). L-Thyroxine improves vestibular compensation in a rat model of acute peripheral vestibulopathy: Cellular and behavioral aspects.. Cells.

[134] El Mahmoudi N., Marouane E., Rastoldo G., Pericat D., Watabe I., Lapotre A., Tonetto A., Chabbert C., Tighilet B. (2022). Microglial dynamics modulate vestibular compensation in a rodent model of vestibulopathy and condition the expression of plasticity mechanisms in the deafferented vestibular nuclei.. Cells.

[135] Wang J., Liu B., Sun F., Xu Y., Luan H., Yang M., Wang C., Zhang T., Zhou Z., Yan H. (2022). Histamine H3R antagonist counteracts the impaired hippocampal neurogenesis in Lipopolysaccharide-induced neuroinflammation.. Int. Immunopharmacol..

[136] Saraiva C., Barata-Antunes S., Santos T., Ferreiro E., Cristóvão A.C., Serra-Almeida C., Ferreira R., Bernardino L. (2019). Histamine modulates hippocampal inflammation and neurogenesis in adult mice.. Sci. Rep..

[137] Frejo L., Lopez-Escamez J.A. (2022). Cytokines and inflammation in meniere disease.. Clin. Exp. Otorhinolaryngol..

[138] Chabbert C. (2016). Principles of vestibular pharmacotherapy.. Handb. Clin. Neurol..

[139] Zwergal A., Strupp M., Brandt T. (2019). Advances in pharmacotherapy of vestibular and ocular motor disorders.. Expert Opin. Pharmacother..

[140] Lacour M., Sterkers O. (2001). Histamine and betahistine in the treatment of vertigo: Elucidation of mechanisms of action.. CNS Drugs.

[141] Lin E., Aligene K. (2013). Pharmacology of balance and dizziness.. NeuroRehabilitation.

[142] Soto E., Vega R., Seseña E. (2013). Neuropharmacological basis of vestibular system disorder treatment.. J. Vestib. Res..

[143] Venail F., Attali P., Wersinger E., Gomeni R., Poli S., Schmerber S. (2018). Safety, tolerability, pharmacokinetics and pharmacokinetic‐pharmacodynamic modelling of the novel H 4 receptor inhibitor SENS‐111 using a modified caloric test in healthy subjects.. Br. J. Clin. Pharmacol..

[144] Fermin H., Van Deinse J.B., Hammelburg E. (1950). The effect of dimenhydrinate upon the labyrinth; (an experimental study).. Acta Otolaryngol..

[145] Halpert A., Olmstead M.C., Beninger R.J. (2002). Mechanisms and abuse liability of the anti-histamine dimenhydrinate.. Neurosci. Biobehav. Rev..

[146] Jaju B.P., Wang S.C. (1971). Effects of diphenhydramine and dimenhydrinate on vestibular neuronal activity of cat: A search for the locus of their antimotion sickness action.. J. Pharmacol. Exp. Ther..

[147] Kirtane M.V., Bhandari A., Narang P., Santani R. (2019). Cinnarizine: A contemporary review.. Indian J. Otolaryngol. Head Neck Surg..

[148] Mangabeira-Albernaz P.L., Ganança M.M., Novo N.F., de Paiva E.R. (1978). Flunarizine and cinnarizine as vestibular depressants. A statistical study.. ORL J. Otorhinolaryngol. Relat. Spec..

[149] Haasler T., Homann G., Duong D.T.A., Jüngling E., Westhofen M., Lückhoff A. (2009). Pharmacological modulation of transmitter release by inhibition of pressure-dependent potassium currents in vestibular hair cells.. Naunyn Schmiedebergs Arch. Pharmacol..

[150] Overstall P.W., Hazell J.W.P., Johnson A.L. (1981). Vertigo in the elderly.. Age Ageing.

[151] Ehlert F.J., Yamamura H.I. (1984). A comparison of the effects of cinnarizine and related compounds on [3H]nitrendipine binding in the brain, heart and ileum.. Life Sci..

[152] Fujimoto S., Sasa M., Takaori S., Matsuoka I. (1978). Selective effect of cinnarizine on the vestibular nucleus neurons.. Arch. Otorhinolaryngol..

[153] Hahn A., Novotný M., Shotekov P.M., Cirek Z., Bognar-Steinberg I., Baumann W. (2011). Comparison of cinnarizine/dimenhy-drinate fixed combination with the respective monotherapies for vertigo of various origins: A randomized, double-blind, active-controlled, multicentre study.. Clin. Drug Investig..

[154] Scholtz A.W., Ilgner J., Loader B., Pritschow B.W., Weisshaar G. (2016). Cinnarizine and dimenhydrinate in the treatment of vertigo in medical practice.. Wien. Klin. Wochenschr..

[155] Plescia F., Salvago P., Dispenza F., Messina G., Cannizzaro E., Martines F. (2021). Efficacy and pharmacological appropriateness of cinnarizine and dimenhydrinate in the treatment of vertigo and related symptoms.. Int. J. Environ. Res. Public Health.

[156] Scholtz A.W., Hahn A., Stefflova B., Medzhidieva D., Ryazantsev S.V., Paschinin A., Kunelskaya N., Schumacher K., Weisshaar G. (2019). Efficacy and safety of a fixed combination of cinnarizine 20 mg and dimenhydrinate 40 mg *vs* betahistine dihydrochloride 16 mg in patients with peripheral vestibular vertigo: A prospective, multinational, multicenter, double-blind, randomized, non-inferiority clinical trial.. Clin. Drug Investig..

[157] Scholtz A.W., Waldfahrer F., Hampel R., Weisshaar G. (2022). Efficacy and safety of a fixed-dose combination of cinnarizine 20 mg and dimenhydrinate 40 mg in the treatment of patients with vestibular vertigo: An individual patient data meta-analysis of randomised, double-blind, controlled clinical trials.. Clin. Drug Investig..

[158] Taghdiri F., Togha M., Razeghi J.S., Refaeian F. (2014). Cinnarizine for the prophylaxis of migraine associated vertigo: A retrospective study.. Springerplus.

[159] Teggi R., Colombo B., Gatti O., Comi G., Bussi M. (2015). Fixed combination of cinnarizine and dimenhydrinate in the prophylactic therapy of vestibular migraine: An observational study.. Neurol. Sci..

[160] Corvera J., Corvera-Behar G., Lapilover V., Ysunza A. (2002). Objective evaluation of the effect of flunarizine on vestibular neuritis.. Otol. Neurotol..

[161] Rashid S.M.U., Sumaria S., Koohi N., Arshad Q., Kaski D. (2022). Patient experience of flunarizine for vestibular migraine: Single centre observational study.. Brain Sci..

[162] Yiannakis C., Hamilton L., Slim M., Kontorinis G. (2023). A systematic review and meta-analysis of prophylactic medication of vestibular migraine.. J. Laryngol. Otol..

[163] Jeck-Thole S., Wagner W. (2006). Betahistine.. Drug Saf..

[164] Murdin L., Hussain K., Schilder A.G. (2016). Betahistine for symptoms of vertigo.. Cochrane Database Syst. Rev..

[165] Bertlich M., Ihler F., Sharaf K., Weiss B.G., Strupp M., Canis M. (2014). Betahistine metabolites, aminoethylpyridine, and hydroxyethylpyridine increase cochlear blood flow in guinea pigs *in vivo*.. Int. J. Audiol..

[166] Ihler F., Bertlich M., Sharaf K., Strieth S., Strupp M., Canis M. (2012). Betahistine exerts a dose-dependent effect on cochlear stria vascularis blood flow in guinea pigs *in vivo*.. PLoS One.

[167] Laurikainen E.A., Miller J.M., Quirk W.S., Kallinen J., Ren T., Nuttall A.L., Grénman R., Virolainen E. (1993). Betahistine-induced vascular effects in the rat cochlea.. Am. J. Otol..

[168] Martinez D.M. (1972). The effect of Serc (betahistine hydrochloride) on the circulation of the inner ear in experimental animals.. Acta Otolaryngol..

[169] Bertlich M., Ihler F., Freytag S., Weiss B.G., Strupp M., Canis M. (2015). Histaminergic H-3-heteroreceptors as a potential mediator of betahistine-induced increase in cochlear blood flow.. Audiol. Neurotol..

[170] Bertlich M., Ihler F., Weiss B.G., Freytag S., Strupp M., Jakob M., Canis M. (2017). Role of capillary pericytes and precapillary arterioles in the vascular mechanism of betahistine in a guinea pig inner ear model.. Life Sci..

[171] Dziadziola J.K., Laurikainen E.L., Rachel J.D., Quirk W.S. (1999). Betahistine increases vestibular blood flow.. Otolaryngol. Head Neck Surg..

[172] Laurikainen E., Miller J.M., Quirk W.S., Nuttall A.L. (1998). The vascular mechanism of action of betahistine in the inner ear of the guinea pig.. Eur. Arch. Otorhinolaryngol..

[173] Dyhrfjeld-Johnsen J., Attali P. (2019). Management of peripheral vertigo with antihistamines: New options on the horizon.. Br. J. Clin. Pharmacol..

[174] Adrion C., Fischer C.S., Wagner J., Gürkov R., Mansmann U., Strupp M. (2016). Efficacy and safety of betahistine treatment in patients with Meniere’s disease: Primary results of a long term, multicentre, double blind, randomised, placebo controlled, dose defining trial (BEMED trial).. BMJ.

[175] Lezius F., Adrion C., Mansmann U., Jahn K., Strupp M. (2011). High-dosage betahistine dihydrochloride between 288 and 480 mg/day in patients with severe Menière’s disease: a case series.. Eur. Arch. Otorhinolaryngol..

[176] Liu J.L., Liu J.G., Chen X.B., Liu Y.H. (2020). The benefits of betahistine or vestibular rehabilitation (Tetrax biofeedback) on the quality of life and fall risk in patients with Ménière’s disease.. J. Laryngol. Otol..

[177] Nauta J.J.P. (2014). Meta-analysis of clinical studies with betahistine in Ménière’s disease and vestibular vertigo.. Eur. Arch. Otorhinolaryngol..

[178] Ramos A.R., Ledezma R.J.G., Navas R. (2015). A.; Cardenas Nuñez, J.L.; Rodríguez, M.V.; Deschamps, J.J.; Liviac, T.J.A. Use of betahistine in the treatment of peripheral vertigo.. Acta Otolaryngol..

[179] Sanchez-Vanegas G., Castro-Moreno C., Buitrago D. (2020). Betahistine in the treatment of peripheral vestibular vertigo: Results of a real-life study in primary care.. Ear Nose Throat J..

[180] Van Esch B., van der Zaag-Loonen H., Bruintjes T., van Benthem P.P. (2022). Betahistine in ménière’s disease or syndrome: A systematic review.. Audiol. Neurotol..

[181] Tighilet B., Leonard J., Lacour M. (1995). Betahistine dihydrochloride treatment facilitates vestibular compensation in the cat.. J. Vestib. Res..

[182] Fukuda J., Matsuda K., Sato G., Kitahara T., Matsuoka M., Azuma T., Kitamura Y., Tomita K., Takeda N. (2021). Effects of betahistine on the development of vestibular compensation after unilateral labyrinthectomy in rats.. Brain Sci..

[183] Zhang Y.X., Wang H.X., Li Q.X., Chen A.X., Wang X.X., Zhou S., Xie S.T., Li H.Z., Wang J.J., Zhang Q., Zhang X.Y., Zhu J.N. (2022). A comparative study of vestibular improvement and gastrointestinal effect of betahistine and gastrodin in mice.. Biomed. Pharmacother..

[184] Tian C.J., Kim S.W., Kim Y.J., Lim H.J., Park R., So H.S., Choung Y.H. (2013). Red ginseng protects against gentamicin-induced balance dysfunction and hearing loss in rats through antiapoptotic functions of ginsenoside Rb1.. Food Chem. Toxicol..

[185] Tighilet B., Trico J., Xavier F., Chabbert C. (2022). What predictability for animal models of peripheral vestibular disorders?. Biomedicines.

[186] Eisenman D.J., Speers R., Telian S.A. (2001). Labyrinthectomy versus vestibular neurectomy: Long-term physiologic and clinical outcomes.. Otol. Neurotol..

[187] Hoffmann K.K., Silverstein H. (2003). Inner ear perfusion: Indications and applications.. Curr. Opin. Otolaryngol. Head Neck Surg..

[188] Sargent E.W., Liao E., Gonda R.L. (2016). Cochlear patency after transmastoid labyrinthectomy for ménière’s syndrome.. Otol. Neurotol..

[189] Van de Heyning P., Betka J., Chovanec M., Devèze A., Giannuzzi A.L., Krempaská S., Przewoźny T., Scheich M., Strupp M., Van Rompaey V., Meyer T. (2023). Efficacy and safety of intranasal betahistine in the treatment of surgery-induced acute vestibular syndrome: a double-blind, randomized, placebo-controlled phase 2 Study.. Otol. Neurotol..

[190] Arrang J.M., Garbarg M., Schwartz J.C. (1983). Auto-inhibition of brain histamine release mediated by a novel class (H3) of histamine receptor.. Nature.

[191] Arrang J.M., Garbarg M., Schwartz J.C. (1985). Autoregulation of histamine release in brain by presynaptic H3-receptors.. Neuroscience.

[192] Arrang J-M., Garbarg M., Schwartz J-C. (1987). Autoinhibition of histamine synthesis mediated by presynaptic H3-receptors.. Neuroscience.

[193] Schwartz J.C., Arrang J.M., Garbarg M., Gulat-Marnay C., Pollard H. (1990). Modulation of histamine synthesis and release in brain *via* presynaptic autoreceptors and heteroreceptors.. Ann. N. Y. Acad. Sci..

[194] Bergquist F., Ruthven A., Ludwig M., Dutia M.B. (2006). Histaminergic and glycinergic modulation of GABA release in the vestibular nuclei of normal and labyrinthectomised rats.. J. Physiol..

[195] Bergquist F., Dutia M.B. (2006). Central histaminergic modulation of vestibular function - a review.. Sheng Li Xue Bao.

[196] Tighilet B., Trottier S., Lacour M. (2005). Dose- and duration-dependent effects of betahistine dihydrochloride treatment on histamine turnover in the cat.. Eur. J. Pharmacol..

[197] Chávez H., Vega R., Valli P., Mira E., Benvenuti C., Guth P.S., Soto E. (2001). Action mechanism of betahistine in the vestibular end organs.. Acta Otorhinolaryngol. Ital..

[198] Soto E., Chávez H., Valli P., Benvenuti C., Vega R. (2001). Betahistine produces post-synaptic inhibition of the excitability of the primary afferent neurons in the vestibular endorgans.. Acta Otolaryngol. Suppl..

[199] Bellot-Saez A., Kékesi O., Morley J.W., Buskila Y. (2017). Astrocytic modulation of neuronal excitability through K + spatial buffering.. Neurosci. Biobehav. Rev..

[200] Losi G., Mariotti L., Sessolo M., Carmignoto G. (2017). New tools to study astrocyte ca2+ signal dynamics in brain networks *in vivo*.. Front. Cell. Neurosci..

[201] Jurič D.M., Kržan M., Lipnik-Stangelj M. (2016). Histamine and astrocyte function.. Pharmacol. Res..

[202] Kárpáti A., Yoshikawa T., Nakamura T., Iida T., Matsuzawa T., Kitano H., Harada R., Yanai K. (2018). Histamine elicits glutamate release from cultured astrocytes.. J. Pharmacol. Sci..

[203] Fang Q., Hu W.W., Wang X.F., Yang Y., Lou G.D., Jin M.M., Yan H.J., Zeng W.Z., Shen Y., Zhang S.H., Xu T.L., Chen Z. (2014). Histamine up-regulates astrocytic glutamate transporter 1 and protects neurons against ischemic injury.. Neuropharmacology.

[204] Ferrini F., De Koninck Y. (2013). Microglia control neuronal network excitability *via* BDNF signalling.. Neural Plast..

[205] Hagemeyer N., Hanft K.M., Akriditou M.A., Unger N., Park E.S., Stanley E.R., Staszewski O., Dimou L., Prinz M. (2017). Microglia contribute to normal myelinogenesis and to oligodendrocyte progenitor maintenance during adulthood.. Acta Neuropathol..

[206] Botta L., Mira E., Valli S., Zucca G., Perin P., Benvenuti C., Fossati A., Valli P. (2000). Effects of betahistine metabolites on frog ampullar receptors.. Acta Otolaryngol..

[207] Qin D., Zhang H., Wang J., Hong Z. (2019). Histamine H4 receptor gene polymorphisms: a potential contributor to Meniere disease.. BMC Med. Genomics.

[208] Zampeli E., Tiligada E. (2009). The role of histamine H 4 receptor in immune and inflammatory disorders.. Br. J. Pharmacol..

[209] Gazquez I., Soto-Varela A., Aran I., Santos S., Batuecas A., Trinidad G., Perez-Garrigues H., Gonzalez-Oller C., Acosta L., Lopez-Escamez J.A. (2011). High prevalence of systemic autoimmune diseases in patients with Menière’s disease.. PLoS One.

[210] Zhou P., Homberg J.R., Fang Q., Wang J., Li W., Meng X., Shen J., Luan Y., Liao P., Swaab D.F., Shan L., Liu C. (2019). Histamine-4 receptor antagonist JNJ7777120 inhibits pro-inflammatory microglia and prevents the progression of Parkinson-like pathology and behaviour in a rat model.. Brain Behav. Immun..

